# Polymorphisms on 8q24 Are Associated with Lung Cancer Risk and Survival in Han Chinese

**DOI:** 10.1371/journal.pone.0041930

**Published:** 2012-07-27

**Authors:** Xuelin Zhang, Qun Chen, Chunya He, Weihua Mao, Ling Zhang, Xiaowen Xu, Junfei Zhu, Baofu Chen

**Affiliations:** 1 Department of Thoracic Surgery, Taizhou Central Hospital, Taizhou, Zhejiang, China; 2 Department of Oncology, Fuzhou Pulmonary Hospital, Fujian Medical University, Fuzhou, Fujian, China; 3 Department of Surgical Oncology, Taizhou Central Hospital, Taizhou, Zhejiang, China; 4 Department of Gynaecology and Obstetrics, Taizhou Central Hospital, Taizhou, Zhejiang, China; 5 Department of Respiratory Medicine, Taizhou Central Hospital, Taizhou, Zhejiang, China; 6 Department of Thoracic Surgery, Taizhou Hospital, Taizhou, Zhejiang, China; University of Aberdeen, United Kingdom

## Abstract

Chromosome 8q24 is commonly amplified in many types of cancer, particularly lung cancer. Polymorphisms in this region are associated with risk of different cancers. To investigate the relationship between three single nucleotide polymorphisms (SNPs) (rs1447295, rs16901979 and rs6983267) on 8q24 and lung cancer risk, we conducted an association study in two Han Chinese populations: one population was from Zhejiang Province (576 case patients and 576 control subjects), whereas the other was from Fujian Province (576 case patients and 576 control subjects). We found that rs6983267 was significantly associated with an increased risk of lung cancer in both populations. Compared with the TT genotype, the GG genotype was associated with a significant 1.555-fold increased risk of lung cancer [95% confidence interval (CI) 1.218–1.986, *P* = 4.0×10^−4^]. This effect was more pronounced in never-smokers [odds ratio (OR) = 2.366, 95% CI 1.605–3.488, *P* = 1.4×10^−5^]. Analyses stratified by histology revealed that rs6983267 GG genotype was most associated with patients with other histological types (OR = 3.012, 95% CI 1.675–5.417, *P* = 2.3×10^−4^). The AA genotype of rs1447295 was associated with increased risk for adenocarcinoma compared with the CC genotype (OR = 2.260, 95% CI 1.174–4.353, *P* = 0.015). Furthermore, the GG genotype of rs6983267 was associated with worse survival in the Zhejiang population (hazard ratio (HR) = 1.646, 95% CI 1.099–2.464, *P* = 0.016). No association was observed for rs16901979. These results suggest that genetic variations on 8q24 may play significant roles in the development and progression of lung cancer.

## Introduction

Lung cancer continues to be the leading cause of cancer-related death in both men and women worldwide. In China, lung cancer accounted for 536,407 new cases of cancer and 475,768 deaths registered in 2005 [1]. In recent decades, the incidence and mortality rates of lung cancer have markedly increased due to the aging of the population and continuous increase in tobacco consumption in China [1], [2], [3], [4]. Although cigarette smoking is the major cause of lung cancer and contributes to more than 80% of cases [5], only a small fraction of smokers develop lung cancer. This finding indicates that genetic factors also affect the risk of lung cancer. Although efforts have been made to identify the genetic factors for lung cancer, current knowledge to assess lung cancer risk remains limited.

Recently, some genome-wide association studies (GWAS) have found that variants on chromosome 8q24 are related to the risk of several cancers, including breast, prostate, bladder, colorectal, and ovarian cancers, among multiple study populations [6], [7], [8], [9], [10], [11], [12], [13], [14], [15], [16], [17]. In addition, variants on 8q24 have been found to be associated with susceptibility to upper gastrointestinal cancer [18], [19], [20], osteosarcoma [21], thyroid cancer [22], [23], [24], and brain cancer [25]. This region spans approximately 600 kb and is considered as a gene-poor area, with relatively few predicted genes, including DQ486513, CB104826, and DQ515897, as well as pseudogene POU5F1P1. The proto-oncogene v-myc myelocytomatosis viral oncogene homolog (avian) (MYC) has been determined to be the cancer-related gene closest to the region associated with cancer in GWAS. Transcriptional enhancers in this region have been demonstrated to physically interact with the MYC promoter [26], [27] and potentially be affected by cancer-associated variants [28].

Three adjacent genomic blocks (regions 1–3) on 8q24 have been associated with prostate cancer risk [29]. The most significant single-nucleotide polymorphisms (SNPs) are rs1447295 in region 1, rs16901979 in region 2, and rs6983267 in region 3 [29]. These SNPs and additional 8q24 variants have been validated by subsequent association studies. Moreover, these SNPs have been found to be associated with other cancers [8], [9], [18], [19], [20], [21], [30]. Since the 8q24 chromosomal region is commonly amplified in lung cancer tissues [31], [32], [33], [34], genetic variants on 8q24 may be associated with lung cancer susceptibility. Furthermore, SNPs on 8q24 may affect the expression levels of cancer-related genes [26], [27], [35], which are believed to play roles in lung cancer carcinogenesis. In the present study, we selected one SNP from each region to evaluate the potential association of these SNPs with lung cancer in Han Chinese.

## Results

### Population characteristics

The characteristics of the lung cancer patients and control subjects enrolled in this study are summarized in Table 1. More men than women were included among the case patients and control subjects, but sex differences between these groups were not significant (*P*>0.05), suggesting their adequate matching. The average age of the population was 58.2 years, and no significant difference in age was found between controls and lung cancer patients (*P*>0.05). Histological type of lung cancer between two patient groups did not significantly differ (*P*>0.05), but significant differences in stage and grade were detected (all *P*<0.05).

**Table 1:** Characteristic of cases and controls.

Characterristic	Population 1			Population 2			Overall		
	Cases (%)	Controls (%)	*P*	Cases (%)	Controls (%)	*P*	Cases (%)	Controls (%)	*P*
**Total**	548	556		453	507		1001	1063	
**Age (years)**									
Median (25% quartile B75% quartile)	58 (51–64)	58 (52–64)	0.756	58 (52–64)	59 (53–65)	0.148	58 (52–64)	58 (52–65)	0.079
**Gender**									
Male	376 (68.6)	389 (70.0)		323 (71.3)	339 (66.9)		699 (69.8)	728 (68.5)	
Female	172 (31.4)	167 (30.0)	0.627	130 (28.7)	168 (33.1)	0.138	302 (30.2)	335 (31.5)	0.509
**Smoking status**									
Never	223 (41.0)	283 (52.9)		157 (34.7)	256 (50.6)		380 (38.1)	539 (51.8)	
Current	231 (42.5)	203 (37.9)		178 (39.3)	164 (32.4)		409 (41.0)	367 (35.3)	
Former	86 (15.8)	43 (8.0)		66 (14.6)	47 (9.3)		152 (15.2)	90 (8.6)	
Unknown	4 (0.7)	6 (1.1)	<0.001	52 (11.5)	39 (7.7)	<0.001	56 (5.6)	45 (4.3)	<0.001
**Stage at diagnosis**									
I	135 (24.6)			83 (18.3)			218 (21.8)		
II	99 (18.1)			63 (13.9)			162 (16.2)		
III	172 (31.4)			190 (41.9)			362 (36.2)		
IV	111 (20.3)			113 (24.9)			224 (22.4)		
Missing	31 (5.7)			4 (0.9)			35 (3.5)		
**Grade**									
Grade 1 +2	336 (61.3)			253 (55.8)			589 (58.8)		
Grade 3	167 (30.5)			195 (43.0)			362 (36.2)		
Missing	45 (8.2)			5 (1.1)			50 (5.0)		
**Histology**									
Squamous cell carcinoma	129 (23.5)			113 (24.9)			242 (24.2)		
Adenocarcinoma	348 (63.5)			298 (65.8)			646 (64.5)		
Adenosquamous	13 (2.4)			10 (2.2)			23 (2.3)		
Others	58 (10.6)			32 (7.1)			90 (9.0)		

### Association analysis of rs1447295, rs16901979, and rs6983267 on 8q24

The genotype distributions of the SNPs rs1447295, rs16901979, and rs6983267 were in Hardy–Weinberg equilibrium (HWE) in both cases and controls. In the test set, the frequency of the rs6983267 GG genotype was markedly higher in lung cancer patients (24.9%) than in control subjects (20.5%) (Table 2). The GG genotype was associated with a modestly increased risk [odds ratio (OR) = 1.532, 95% confidence interval (CI) 1.090–2.153, *P* = 0.014]. Similarly, in the validation set, the rs6983267 GG genotype was associated with a 1.591-fold increase in the risk of lung cancer (*P* = 0.010). Although not statistically significant, the rs6983267 GT genotype also increased the risk of lung cancer (*P* = 0.062). However, there were no significant differences between the remaining two SNPs (rs1447295 and rs16901979) and lung cancer in both populations. The final pooled analysis showed that the rs6983267 GG genotype (OR = 1.555, 95% CI = 1.218–1.986, *P* = 4.0×10^−4^) and GT genotype (OR = 1.311, 95% CI = 1.066–1.613, *P* = 0.01) were associated with significantly increased risk of lung cancer. After the GG and GT genotypes were combined, the difference remained statistically significant (OR = 1.386, 95% CI = 1.141–1.684, *P* = 0.001). Moreover, the associations remained significant even after Bonferroni correction (i.e., *P*<0.05/3 = 0.0167).

**Table 2:** Association between three SNPs in 8q24 and risk of lung cancer.

SNP	Genotype	Test set (548/556)			Validation set (453/507)			Pooled (1001/1063)		
		No.^a^	OR (95% CI)^b^	*P* b	No.^a^	OR (95% CI)^b^	*P* b	No.^a^	OR (95% CI)^b^	*P* b
rs1447295	CC	406/407	Reference		323/381	Reference		729/788	Reference	
	AC	129/139	0.933 (0.708–1.231)	0.625	116/118	1.166 (0.866–1.568)	0.312	245/257	1.033 (0.844–1.264)	0.756
	AA	13/10	1.336 (0.578–3.087)	0.498	14/8	2.172 (0.896–5.268)	0.086	27/18	1.678 (0.915–3.079)	0.094
	*P* value for trend			0.782			0.079			0.167
rs16901979	CC	263/285	Reference		209/248	Reference		472/533	Reference	
	AC	237/228	1.128 (0.880–1.444)	0.342	208/215	1.152 (0.883–1.502)	0.298	445/443	1.138 (0.950–1.364)	0.162
	AA	48/43	1.215 (0.779–1.896)	0.39	36/44	0.963 (0.596–1.554)	0.876	84/87	1.091 (0.789–1.510)	0.598
	*P* value for trend			0.339			0.655			0.321
rs6983267	TT	135/173	Reference		115/164	Reference		250/337	Reference	
	GT	276/269	1.285 (0.967–1.708)	0.084	214/231	1.335 (0.986–1.809)	0.062	490/500	1.311 (1.066–1.613)	0.010
	GG	136/114	1.532 (1.090–2.153)	0.014	123/112	1.591 (1.118–2.264)	0.010	259/226	1.555 (1.218–1.986)	4.0×10^−4^
	*P* value for trend			0.013			0.009			3.4×10^−4^

a cases/controls.

b adjusted for age, sex and smoking status.

The analyses stratified by histology using pooled case-control sets showed that the rs1447295 AA genotype as well as the rs6983267 GG and GT genotypes were associated with a weak but significant increase in the risk of adenocarcinoma adjusted for age, sex, and smoking status (OR = 2.260, 95% CI 1.174–4.353, *P* = 0.015; OR = 1.330, 95% CI 1.003–1.763, *P* = 0.048; OR = 1.327, 95% CI 1.049–1.679, *P* = 0.018, respectively) (Table 3). The rs6983267 GG genotype was associated with squamous cell carcinoma risk (OR = 1.775, 95% *CI* 1.201–2.625, *P* = 0.004). In addition, individuals with the rs6983267 GG or GT genotype were at significantly increased risk of other types of lung cancer (OR = 3.012, 95% CI 1.675–5.417, *P* = 2.3×10^−4^; OR = 1.986, 95% CI 1.157–3.408, *P* = 0.013, respectively), including adenosquamous carcinoma and undifferentiated carcinoma. However, no association was observed for rs16901979.

**Table 3:** Genotype distribution of three SNPs by histology in patients and controls.

SNP	Genotype	Adenocarcinoma			Squamous cell carcinoma			Others		
		No.^a^	OR (95% CI)^b^	*P* b	No.^a^	OR (95% CI)^b^	*P* b	No.^a^	OR (95% CI)^b^	*P* b
rs1447295	CC	451/788	Reference		192/788	Reference		82/788	Reference	
	AC	173/257	1.243 (0.989–1.563)	0.063	44/257	0.716 (0.496–1.035)	0.076	28/257	1.048 (0.665–1.651)	0.840
	AA	22/18	2.260 (1.174–4.353)	0.015	2/18	0.555 (0.124–2.480)	0.440	3/18	1.781 (0.502–6.311)	0.371
	*P* value for trend			0.005			0.057			0.427
rs16901979	CC	295/533	Reference		118/533	Reference		56/533	Reference	
	AC	286/443	1.189 (0.965–1.465)	0.103	109/443	1.088 (0.805–1.469)	0.584	49/443	1.053 (0.704–1.573)	0.803
	AA	65/87	1.350 (0.946–1.926)	0.098	11/87	0.546 (0.279–1.069)	0.077	8/87	0.678 (0.297–1.543)	0.354
	*P* value for trend			0.041			0.396			0.747
rs6983267	TT	165/337	Reference		66/337	Reference		19/337	Reference	
	GT	328/500	1.327 (1.049–1.679)	0.018	100/500	1.018 (0.718–1.445)	0.919	58/500	1.986 (1.157–3.408)	0.013
	GG	151/226	1.330 (1.003–1.763)	0.048	72/226	1.775 (1.201–2.625)	0.004	36/226	3.012 (1.675–5.417)	2.3×10^−4^
	*P* value for trend			0.035			0.006			1.9×10^−4^

a cases/controls.

b adjusted for age, gender and smoking status.

We further examined the association between the three SNPs and lung cancer stratified by smoking behavior in all individuals (Table 4). The rs6983267 GG genotype was significantly associated with higher risk of lung cancer in never-smokers, but no association was observed in former and current-smokers. Moreover, there was a slight but significant difference in frequency of rs6983267 GT genotype between cases and controls in current-smokers (OR = 1.446, 95% CI 1.032–2.027, *P* = 0.032). However, there was no significant interaction between smoking status and genotype in the lung cancer susceptibility. Since the number of former-smokers in this study was small and the frequency of the rs1447295 AA genotype was low, we combined the AA and AC genotype groups into one. Logistic analysis revealed that AA and AC combined had a lower risk of lung cancer than the CC genotype (OR = 0.478, 95% CI 0.254–0.898, *P* = 0.022). No other significant association was observed between rs16901979 and lung cancer risk, stratified by smoking status.

**Table 4:** Genotype distribution of three SNPs by smoking status in patients and controls.

SNP	Genotype	Never			Current			Former		
		No.^a^	OR (95% CI)^b^	*P* b	No.^a^	OR (95% CI)^b^	*P* b	No.^a^	OR (95% CI)^b^	*P* b
rs1447295	CC	275/407	Reference		295/278	Reference		121/61	Reference	
	AC	95/122	1.127 (0.820–1.548)	0.461	104/82	1.173 (0.840–1.639)	0.349	28/29	0.478 (0.254–0.898)^c^	0.022^c^
	AA	10/10	1.588 (0.623–4.045)	0.333	10/7	1.463 (0.543–3.942)	0.451	3/0		
	*P* value for trend			0.270			0.249			0.022
rs16901979	CC	175/283	Reference		197/181	Reference		79/41	Reference	
	AC	168/207	1.288 (0.968–1.715)	0.083	184/154	1.122 (0.834–1.510)	0.446	60/46	0.674 (0.380–1.193)	0.175
	AA	37/49	1.049 (0.654–1.682)	0.843	28/32	0.803 (0.464–1.390)	0.433	13/3	2.650 (0.660–10.640)	0.169
	*P* value for trend			0.302			0.937			0.991
rs6983267	TT	95/170	Reference		100/118	Reference		42/27	Reference	
	GT	174/274	1.160 (0.841–1.600)	0.366	203/167	1.446 (1.032–2.027)	0.032	74/40	1.509 (0.782–2.912)	0.220
	GG	114/95	2.366 (1.605–3.488)	1.4×10^−5^	104/82	1.430 (0.962–2.126)	0.077	36/23	1.180 (0.558–2.495)	0.665
	*P* value for trend			2.8×10^−5^			0.063			0.498

a cases/controls.

b adjusted for age and gender.

c AA+AC vs CC.

### Association of rs1447295, rs16901979, and rs6983267 with survival

**Figure 1:**
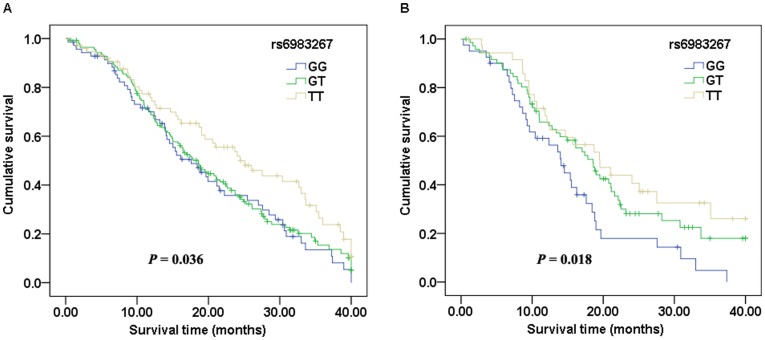
Kaplan–Meier survival curves for lung cancer patients in the Zhejiang study population with different rs6983267 genotypes. A: all lung cancer patients. B: patients with stage III–IV tumors.

All patients with lung cancer were included in the survival analysis. For rs6983267, an association with lung cancer survival was observed in the Zhejiang population. Individuals with the GG genotype had a median survival time (MST) of 17.6 months, those with the GT genotype had an MST of 18.6 months, and those with the TT genotype had an MST of 24.4 months (log-rank test, *P* = 0.036) (Figure 1A). In the Cox proportional hazards model, we found that the hazard ratio (HR) was significantly higher for individuals with the GG genotype compared with those with the TT genotype after adjusted for age, sex, smoking status, tumor stage, histology and histological grade (HR = 1.646, 95% CI 1.099–2.464, *P* = 0.016) (Table 5). The survival was poor especially for patients with stage III or IV tumor (HR = 1.993, 95% CI 1.128–3.521, *P* = 0.018) (Figure 1B). No other SNP was found to be associated with survival. No association with survival in the Fujian population was observed.

**Table 5:** Association between three SNPs on 8q24 and survival in patients with lung cancer.

SNP	Genotype	Zhejiang population				Fujian population			
		No. of patients	No. of death (%)	HR (95% CI)^a^	*P* a	No. of patients	No. of death (%)	HR (95% CI)^a^	*P* a
rs1447295	CC	209	153 (72.9)	Reference		255	169 (73.2)	Reference	
	AC	70	52 (24.8)	0.983 (0.712–1.358)	0.918	79	53 (22.9)	1.025 (0.751–1.399)	0.877
	AA	6	5 (2.4)	2.272 (0.916–5.634)	0.077	15	9 (3.9)	1.097 (0.548–2.193)	0.794
	Global *P* value				0.201				0.958
rs16901979	CC	139	99 (47.1)	Reference		165	109 (47.2)	Reference	
	AC	119	90 (42.9)	1.079 (0.807–1.444)	0.607	160	105 (45.5)	0.969 (0.739–1.2720	0.823
	AA	27	21 (10.0)	1.041 (0.635–1.705)	0.874	24	17 (7.4)	1.524 (0.907–2.561)	0.111
	Global *P* value				0.876				0.224
rs6983267	TT	75	49 (23.3)	Reference		89	61 (26.4)	Reference	
	GT	140	105 (50.0)	1.411 (0.986–2.018)	0.060	158	110 (47.6)	1.081 (0.750–1.558)	0.675
	GG	70	56 (26.7)	1.646 (1.099–2.464)	0.016	100	60 (26.0)	1.218 (0.884–1.678)	0.227
	Global *P* value				0.049				0.464

a adjusted for age, sex, smoking status, tumor stage, histology and histological grade.

## Discussion

In the present study, we examined whether SNPs on chromosome 8q24 related to susceptibility to breast, prostate, bladder, colorectal, thyroid, and ovarian cancers [6], [7], [8], [9], [10], [11], [12], [13], [14], [15], [16], [23] are also associated with risk of lung cancer in Han Chinese populations. We found that the GG genotype of rs6983267 was significantly associated with increased risk of lung cancer and decreased survival in Han Chinese.

8q24 aberration is a frequent event in lung cancer [31], [32], [33], [34]. MYC, localized at 8q24.1, is a well-established oncogene involved in cell growth, proliferation, differentiation, and apoptosis. Deregulated overexpression of MYC is responsible for a wide range of human cancers. The rs6983267 is located 335 kb upstream from the MYC gene. Recent studies have demonstrated that rs6983267 maps to transcription factor 7-like 2 (TCF7L2) motif and forms long-range interactions with MYC [27], [36]. Compared with the T allele of rs6983267, the G allele has been reported to have stronger TCF7L2 binding in vitro and in vivo and to enhance Wnt activity [36], [37]. Furthermore, many studies have shown that the GG genotype of rs6983267 is associated with a significantly higher risk of several cancers [9], [30], [38]. Park et al. [20] reported no association between rs6983267 (OR = 1.02, *P* = 0.80) and lung cancer but found that the GG genotype increased the risk of lung cancer in ever-smokers (OR = 1.45, *P* = 0.024). Wokolorczyk et al. [38] examined 738 lung cancer patients and 1910 control subjects and found that the rs6983267 GG genotype was not significantly associated with risk of lung cancer compared with the TT genotype (OR = 0.98, *P* = 0.9). However, this association has not been assessed in Chinese. In this study, we examined three SNPs (rs1447295, rs16901979 and rs6983267) and found that only rs6983267 was significantly associated with an increased risk of lung cancer in Han Chinese. These findings were different from previous studies in which there were no significant correlations between rs6983267 and lung cancer [20], [38]. Since the effect of a low-penetrance susceptibility gene on disease risk can be modified by other genes as well as by environmental factors, ethnic differences in genetic backgrounds and different risk factors might explain, to some extent, the discrepant results. Furthermore, rs6983267 was most significantly associated with other histological types of lung cancer, followed by squamous cell carcinoma, and weakly but significantly associated with adenocarcinoma. These results indicated that different mechanisms may exist in the molecular pathogenesis of the various histological types of lung cancer.

Exposure to tobacco carcinogens may induce severe DNA damage and thus promote tumor formation. In this study, we found that the increased risk of lung cancer with rs6983267 GG genotype was more pronounced among non-smokers, whereas no significant correlations were found in current- and former-smokers. One possible explanation for this is that never-smokers may be more likely to have been exposed to unknown risk factors associated with lung cancer, such as environmental pollutants. There may exist different carcinogenesis mechanisms between never and current-/former- smokers. Our result is different from previous study, in which rs6983267 GG was a risk factor in ever-smoker [20]. Different study populations and analytical approaches could account for the contradictory results. In addition, we found a significant association of the A allele of rs1447295 with a low risk of lung cancer in former-smokers. However, Park et al found that this allele was associated with a higher risk of liver cancer in ever-smokers [20]. Since the number of former-smokers in this population was very small for a case-control association study, our findings on the A allele of rs1447295 are most likely false positive due to multiple comparisons. Caution should be used in interpreting this result even though it is statistically significant. Further studies with larger numbers of subjects are needed to confirm these findings.

MYC amplification has been shown to be correlated with poor prognosis of patients with lung cancer [31], [32], [34]. Since the risk allele rs6983267 G has been shown to have a stronger affinity to TCF7L2 and may induce enhanced MYC expression [27], [36], rs6983267 might be associated with survival in patients with lung cancer. In this study, we found that rs6983267 GG genotype was associated with markedly worse survival in the Zhejiang population. This effect was more pronounced in patients with stage III or IV tumor. This result is in accordance with the functional consequences of this polymorphism. In contrast, no association was found between rs6983267 and survival in the Fujian population. Part of this disparity may be explained by the distinct percentages of disease stage and histological grade as well as the different treatment approaches used. Cicek et al. examined 393 patients with colon cancer and found no association between rs6983267 and survival [39]. However, Dai et al. examined 170 patients with colorectal cancer and found that T allele was associated with worse survival [40]. These results suggest that a more complex or tumor-specific relationship may exist. As our study was limited to Han Chinese with lung cancer, the results may not be generalizable to other ethnicities. In addition, the sample size for survival analysis was relatively small, thereby prompting cautious interpretation of our results. Further research in a larger population group is necessary to clarify this relationship.

In summary, the findings of our study suggest that the SNPs on 8q24 are associated with susceptibility to lung cancer in Han Chinese. In addition, rs6983267 GG genotype was associated with poor survival in the Zhejiang population. To our knowledge, this is the first study that investigated potential associations between SNPs on 8q24 and lung cancer in a population from East China. Additional large studies are warranted to improve our understanding of the contributions of genetic factors to lung cancer.

## Materials and Methods

### Subjects

In this study, we performed an association study in two independent sets of subjects. The test set included 548 lung cancer patients and 556 control subjects. The case patients had a diagnosis of lung cancer confirmed on histology and were recruited from Taizhou Central Hospital, Zhejiang, between 2003 and 2010. The cancer-free control subjects were hospital visitors who came to the health examination clinic for an annual check-up between 2007 and 2010. The validation set consisted of 453 lung cancer patients and 507 control subjects from Fujian Province. The case patients were consecutive patients with histologically confirmed lung cancer enrolled from Fuzhou Pulmonary Hospital between 2008 and 2010. The control subjects were cancer-free individuals selected from a pool of healthy volunteers who visited the hospital during the period of 2008–2010. Epidemiological data were collected by trained interviewers who were not aware of the group assignments using a specific standardized questionnaire to obtain detailed information on the participants demographic factors, personal habits, medical history, family history of cancer, as well as history of occupational and environmental exposure. Study subjects were classified into three groups based on their smoking status. Never-smokers were those who indicated that they had smoked less than 100 cigarettes in their lifetime and were not current-smokers. Smokers were classified into former smokers and current smokers. Former-smokers were those who reported smoking at least 100 cigarettes during their lifetime and were not current-smokers, whereas current-smokers were those who reported that they were still smoking at the time of their lung cancer diagnosis. Individuals known to have a family history of cancer, a history of any cancer other than lung cancer, or metastasized cancer from other or unknown origins were excluded. The control subjects were frequency matched to the case patients based on sex and age (±5 years). All case patients and control subjects were unrelated ethnic Han Chinese. This study was approved by the ethics committees of Taizhou Central Hospital and Fuzhou Pulmonary Hospital, and written informed consent was obtained from all participants before entering the study.

Our end point was overall survival from the initial histological diagnosis. Patients who were alive or lost to follow-up were censored on the date of last contact. Those who died of other causes were censored on the date of their death. Furthermore, patients lost to follow-up in the first year were excluded from analysis.

### Genotyping

Blood samples were collected from all study participants. Genomic DNA was extracted using Universal Genomic DNA Extraction Kit Version 3.0 (Takara, Dalian, China) according to the manufacturer's protocol, and DNA samples were stored at −20°C.

We selected the strongest single-association SNPs from each chromosomal region: rs1447295 from region 1, rs16901979 from region 2, and rs6983267 from region 3. Genotyping was performed by polymerase chain reaction-ligation detection reaction (PCR-LDR) method. Primers were designed by software of Primer 3 (http://frodo.wi.mit.edu/primer3/input.htm). The three primer pairs are as following: 1) rs1447295: 5′-GCCTACGCCTACTCCTGGTCT-3′ (forward) and 5′-CTCCCAGATTTTCCCATACCC-3′ (reverse); 2) rs16901979: 5′-AGTGTGGGGTCTTTGTTGTGG-3′ (forward) and 5′- CAGCAGTCTCCCTGTCTTTGG-3′ (reverse); 3) rs6983267: 5′- ATGAAGGCGTCGTCCAAATGA -3′ (forward) and 5′- TTGGCTGGCACTGTCTGTATA -3′ (reverse). Amplification reactions were carried out in a final reaction volume of 15 µL containing 0.3 mmol/L of each deoxynucleoside triphosphate, 10 mmol/L Tris-HCl, 50 mmol/L KCl, 2 mmol/L MgCl2, 20% Q solution (Qiagen, Hilden, Germany), 0.16 µmol/L of each primer, 10 ng genomic DNA, and 1 U Taq (TaKaRa, Dalian, China). The cycling parameters were: 94°C for 3 min, followed by 10 cycles of 94°C for 30 s, 64°C for 30 s with a 0.5°C decrement of the annealing temperature per cycle and 72°C for 30 s, followed by 30 cycles of 94°C for 30 s, 59°C for 30 s and 72°C for 30 s, and a final extension step at 72°C for 5 min. After amplification, PCR products were incubated with 2 U shrimp alkaline phosophatase (Fermentas, Vilnius, Lithuania) and 4 U exonuclease I (Fermentas, Vilnius, Lithuania) at 37°C for 1 h, and then denatured at 95°C for 10 min.

The LDR primers were: 1) rs1447295: 5′-GTGCCATTGGGGAGGTATGTAAAAA-3′ (A specific primer), 5′-TTTGTGCCATTGGGGAGGTATGTAAAAC-3′ (C specific primer) and 5′-GTGCTATGGAAAAAAAGCAACAGGA-FAM-3′ (common FAM labeled 3′-end primer); 2) rs16901979: 5′-TTTTTGTTAATGATTTAGCATTACTTATA-3′ (A specific primer), 5′-TTTTTTTTGTTAATGATTTAGCATTACTTATC-3′ (C specific primer) and 5′-TCTGGCAAATGGTATTTTTGAGATATTT-FAM-3′ (common FAM labeled 3′-end primer); 3) rs6983267: 5′-TTTTTTTTTCCTTTGAGCTCAGCAGATGAAAGG-3′ (G specific primer), 5′-TTTTTTTTTTTTCCTTTGAGCTCAGCAGATGAAAGT-3′ (T specific primer) and 5′-CACTGAGAAAAGTACAAAGAATTTTTTTTTTT-FAM-3′ (common FAM labeled 3′-end primer). A mixture containing 7.5 pmol of each common FAM labeled LDR primer was phosphorylated in a 15-µl kinase reaction mixture containing 1× T4 ligase buffer and 10 U of T4 kinase (Takara, Dalian, China). The mixture was incubated at 37°C for 1 h, followed by 10 min at 65°C and storage at 4°C. LDRs were carried out in a final volume of 20 µL including 9 µL of purified PCR product, 1× Taq DNA ligase buffer, 250 fmol of each LDR primer, 8 U Taq DNA ligase (New England Biolabs, Beverly, Mass, USA). The LDR parameters were as follows: 30 cycles of 30 s at 94°C and 4 min at 55°C. The LDR products were analyzed on ABI 3730 DNA analyzer (Applied Biosystems, CA, USA).

Ninety six randomly selected samples were reevaluated by DNA sequencing to confirm the accuracy of the PCR–ligation detection reaction genotyping method, the results of which were consistent with the genotypes identified by direct sequencing. In addition, 96 randomly selected samples were genotyped in duplicate and yielded the same results (100% reproducibility).

### Statistical analysis

The means of quantitative variables were compared using Student's *t* test. Fisher's exact Chi-square test was used to compare categorical variables between case patients and control subjects. HWE was calculated by the Chi-square test for goodness of fit in both patient and control groups. Correction for multiple testing was carried out using the Bonferroni method. To evaluate the relationship between 3 SNPs and the risk of lung cancer, the ORs and their 95% CIs were estimated by multivariate logistic regression analysis, adjusted by age, sex, and smoking status, to evaluate the relationship between each of the three SNPs and the risk of lung cancer. Survival analysis was performed using the Kaplan–Meier method with log-rank test. Cox proportional hazards models were also used to adjust for age, sex, smoking status, tumor stage, histology, and histological grade. *P*<0.05 was considered statistically significant. All tests were two-sided, and all statistical analyses were performed with SPSS 17.0 software package (SPSS Inc, Chicago, USA).
